# A case report of paraproteinemia-associated pauci-immune glomerulonephritis – a new form of monoclonal gammopathy of renal significance? 

**DOI:** 10.5414/CNCS109160

**Published:** 2017-08-17

**Authors:** Robert Rope, Neeraja Kambham, Neiha Arora

**Affiliations:** 1 Division of Nephrology,; 2 Department of Pathology, Stanford University School of Medicine, Stanford, and; 3 Department of Nephrology, Kaiser Permanente, Fremont, CA, USA

**Keywords:** monoclonal gammopathy of unknown significance (MGUS), monoclonal gammopathy of renal significance (MGRS), pauci-immune glomerulonephritis, multiple myeloma

## Abstract

Background: Renal disease associated with paraproteinemias is classically predicated upon pathologic paraprotein deposition in the kidney. However, growing evidence suggests that paraproteins may be able to systemically activate complement or neutrophils to drive renal damage. This may provide an alternative pathologic mechanism for renal injury in rare cases. Case report: We report a case of a patient with crescentic pauci-immune glomerulonephritis presenting with rapidly progressive renal failure, polyarthropathy, and a purpuric rash in association with a monoclonal immunoglobulin G κ-light-chain producing multiple myeloma. Serum anti-neutrophil cytoplasmic antibodies were not detected. Kidney biopsy, including with Pronase digestion, did not reveal pathologic paraprotein deposition. Two previously published similar case reports are also discussed. Conclusion: We propose a novel pathologic mechanism involving monoclonal proteins as a trigger for pauci-immune glomerulonephritis, potentially via complement dysregulation and/or neutrophil activation. This requires further epidemiologic and mechanistic study.

## Introduction 

Over the last two decades, the array of renal pathologies attributed to monoclonal gammopathies has expanded [1]. Proposed disease mechanisms involve the pathologic deposition of paraproteins in the kidney, with detectable immune deposits by kidney biopsy. Here we report a patient with pauci-immune glomerulonephritis (PIGN) and multiple myeloma. This may represent a novel pathogenic mechanism for monoclonal gammopathy-related glomerular disease via antibody-mediated complement or neutrophil activation in the absence of renal paraprotein deposition. 

## Case history 

A 58-year-old Hispanic woman with controlled essential hypertension developed polyarthropathy involving the neck, ankles, wrists, and metacarpal phalangeal joints over several months. She was evaluated by a rheumatologist and diagnosed with seronegative rheumatoid arthritis. She then began treatment with methotrexate and etanercept. Four weeks later, she developed a lacy purpuric rash involving her extremities and torso. She was prescribed prednisone (60 mg/d), and etanercept was discontinued. The rash improved, although worsened when prednisone was tapered. Two months after stopping etanercept and commencing prednisone, methotrexate was switched to oral cyclophosphamide, and the patient was referred to our institution. 

When evaluated 5 days later, the patient was admitted for workup of acute kidney injury with ongoing arthritis and rash. Her serum creatinine had increased to 2.7 mg/dL from 0.8 mg/dL 10 days earlier, and was accompanied by hematuria, proteinuria (1.5 g/24 hours), and worsened hypertension. Cyclophosphamide was discontinued and prednisone continued. 

The patient had no family history of kidney or rheumatologic disease. She did not use tobacco, alcohol, or illicit, herbal, or non-prescription drugs. 

**Figure 1.**
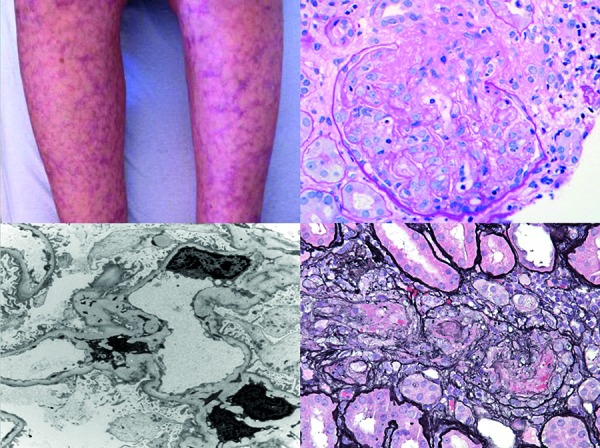
Patient’s rash and kidney biopsy. Upper left: lower extremity rash; upper right: PAS stain showing glomerular necrosis; lower right: silver stain showing arteriolar necrosis; lower left: electron microscopy demonstrating the lack of immunologic deposits.

Initial examination was notable for 1+ bilateral pedal edema, a violet, reticular, non-blanching rash on her face, torso, and extremities, as well as hand and wrist swelling (Figure 1). 

Serologic workup for rapidly progressive glomerulonephritis (RPGN), including testing for anti-neutrophil cytoplasmic antibodies (ANCAs), was notable only for a monoclonal immunoglobulin G (IgG) κ level of 2 g/dL (Table 1). A skeletal survey was negative. Initial left and right pelvic bone marrow biopsies were unrevealing, though one sample was suboptimal. A subsequent PET scan revealed increased uptake in the left iliac crest, sternum, and right clavicle. A third bone marrow biopsy, obtained under fluoroscopic guidance, revealed monotypic κ plasma cells, consistent with multiple myeloma. 

Kidney biopsy revealed PIGN and arteriolar vasculitis (Figure 1). Light microscopy demonstrated necrosis and/or segmental and circumferential cellular and fibrocellular crescents in 6 out of 21 glomeruli. There was no mesangial or endocapillary proliferation. Immunoglobulin deposits were not found by light, immunofluorescence, or electron microscopy. Paraffin immunofluorescence with Pronase digestion, useful in revealing “masked” immune deposits, did not reveal pathologic paraprotein deposition [2]. Mild (1+) segmental mesangial C3 deposition did not fulfill criteria for C3 glomerulopathy [3]. 

On discharge, the patient continued oral prednisone (60 mg/d). Her hypertension and hypervolemia responded to diuretics. Kidney function improved (from a peak serum creatinine of 3.1 mg/dL to 1.4 mg/dL) prior to the initiation of cyclophosphamide, bortezomib, and dexamethasone for multiple myeloma. IgG-κ levels returned to normal with chemotherapy, though the patient’s course was complicated by fatigue, peripheral neuropathy, and venous thromboemboli. Unfortunately, she developed a fatal pulmonary hemorrhage while on systemic anticoagulation. Prior to her death, the patient had completed six cycles of chemotherapy with resolution of PET-avid lesions and further improvement in creatinine to 1.2 mg/dL. 

## Discussion 

This report illustrates an uncommon association between paraproteinemia and crescentic PIGN, which may represent a novel pathogenic mechanism. Kidney disease in the setting of monoclonal gammopathy is common and typically associated with immune deposits by light, immunofluorescence, and/or electron microscopy [1]. The characteristics and serum concentration of the monoclonal protein determine the specific pathologies seen (Table 2). More common pathologies, including cast nephropathy, amyloidosis, and monoclonal immunoglobulin deposition disease, are associated with high tumor burdens and symptomatic disease [1, 4]. These pathologic entities were absent in our case, which is not surprising given her nephritic presentation, the absence of myeloma symptoms, and small tumor burden. Less common pathologies, including heterogeneous forms of proliferative glomerulonephritis, are less dependent on tumor burden and typically present in asymptomatic patients with smaller B-cell clones, such as seen in monoclonal gammopathy of unknown significance (MGUS) [1]. 

MGUS is defined as a known monoclonal protein at less than 3 g/dL with less than 10% plasma cells on biopsy and no end-organ damage. It is a common diagnosis with low malignant potential [5]. However, there is an under-recognized but significant risk for renal damage [6]. In recent years, the importance of this association has been highlighted and the term “monoclonal gammopathy of renal significance” (MGRS) introduced [6]. The diverse disorders that comprise this new entity are believed to result from observable pathologic protein deposition in the kidney. The ultrastructure, location of, and tissue response to these deposits differentiate the disease subtypes. 

In our case, kidney biopsy revealed crescentic PIGN, without immunoglobulin or complement deposition, and without plasma cell infiltration. These findings contrast with the conceptualization of myeloma-related kidney disease as involving direct toxicity from deposited paraproteins. 

Two recent similar case reports highlight the possibility that PIGN may belong in the spectrum of MGRS. Grundmann et al. [7] reported a 60-year-old male with RPGN due to PIGN in the setting of an IgG-κ plasmacytoma and paraproteinemia. The patient’s renal dysfunction improved with bortezomib and dexamethasone. Repeat biopsy after treatment showed resolution of active lesions. Anaele et al. [8] reported a 57-year-old woman presenting with dialysis-dependent renal disease due to sclerosing PIGN also in the setting of an IgG-κ producing plasmacytoma. Unfortunately, this patient’s kidney failure did not improve with chemotherapy. As was the case for our patient, ANCAs were not detected in either case. Notably, a case of myeloperoxidase-ANCA positive crescentic glomerulonephritis in association with IgG-λ myeloma complicated by fatal pulmonary hemorrhage has also been reported [22]. 

While it is possible that immune staining techniques, in our and the prior case reports, were insufficiently sensitive to detect low-level immune deposits, this series of cases raises the possibility that a disease mechanism other than direct toxicity from immune deposition might be involved. In our case, Pronase-aided digestion did not reveal pathologic paraprotein deposits, supporting the idea that such deposits were not present. In addition, though tumor necrosis factor inhibition, including with etanercept, has been linked to PIGN with inconsistent ANCA serologies, the development of RPGN 2 months after stopping etanercept while on immunosuppressive therapy is unlikely [9, 10]. With these considerations, we propose that the monoclonal protein in these cases might activate or dysregulate the complement system, or activate neutrophils by mechanisms independent of ANCA. 

Over the last two decades, there has been a greater appreciation of the role of complement in ANCA-associated vasculitis (AAV) and in paraprotein-associated glomerulonephritis [11]. Complement activation is a key component in the inflammatory amplification loop initiated after ANCAs activate neutrophils [12, 13]. Alternative pathway activation in AAV is supported by the presence of C3a and C5a plasma products that correlate with disease activity [14]. Furthermore, in a mouse model of AAV, blocking the C5a receptor-mediated activation of neutrophils ameliorated renal damage [15]. An oral C5a receptor inhibitor is currently being investigated in humans with AAV [16]. 

Activation of the alternative complement (AC) pathway by monoclonal immunoglobulins has been described in glomerular disease. Immunoglobulins acting as auto-antibodies to Factor H or C3 convertase (C3 nephritic factor) can activate AC resulting in dense-deposit disease [17, 18]. Similarly, monoclonal light chain immunoglobulin has been linked to hypocomplementemic membranoproliferative glomerulonephritis via alternative pathway Factor H inactivation [19]. These examples demonstrate the pathologic potential of paraproteins outside the traditional paradigm of renal deposition and lend theoretical support for the possibility of immunoglobulin-mediated AC activation in pauci-immune AAV. 

The disease process and the role of complement are less well understood for the 10 – 20% of patients with ANCA-negative PIGN [20]. As other autoantibodies have been demonstrated in AAV, (e.g., anti-lysosome-associated membrane protein-2), it is possible that alternative pathologic auto-antibodies exist, for which we do not routinely assay [20]. Perhaps the pathologic antibody activating neutrophils in our case, driving the PIGN, was a myeloma-associated paraprotein. 

These considerations aside, MGUS is present in ~ 3% of individuals over age 50 [5]. While it seems plausible that a monoclonal protein could lead to complement and/or neutrophil activation and thus PIGN, the presence of paraproteins and myeloma in our patient could be coincidental. To further strengthen this potential pathologic relationship, it must be demonstrated that MGUS and/or multiple myeloma are more common than expected in patients with AAV, particularly in ANCA-negative cases [21]. In addition, mechanistic studies evaluating the potential of a paraprotein to activate the pathologic pathways identified in PIGN are needed. 

Importantly, as paraproteinemias are known to cause a wide array of renal pathologies, it appears reasonable to screen most patients with proteinuria and/or hematuria without another clear cause. This may include cases of ANCA-negative PIGN, though the commonplace nature of MGUS must give providers pause. 

In summary, we propose that a pathologic paraprotein resulted in our patient’s ANCA-negative PIGN. Our hypothesis is that the paraprotein itself serves as a trigger, either by systemically activating or dysregulating complement or by systemically activating neutrophils. For similar cases, efforts to reduce paraprotein production should be included as part of any treatment approach. 

## Acknowledgment 

The authors would like to acknowledge and thank the patient and her family for their kindness and consent for publication. They would also like to thank the many healthcare workers who contributed to the patient’s care. 

## Conflict of interest 

The authors have no competing interests or financial conflicts to disclose. 

**Table 1.** Rheumatologic and AKI serologic evaluation.

General/rheumatology workup	Result	Normal
Anti-nuclear antibodies (ANA)	Negative	Negative
Anti-CCP Ab	Negative	Negative
Anti-LA Ab	Negative	Negative
Anti-Ro Ab	Negative	Negative
Anti-RF Ab	Negative	Negative
C3	125	86 – 184 mg/dL
C4	34	20 – 59 mg/dL
Anti-myeloperoxidase Ab	< 0.2	< 0.2 U
Anti-proteinase-3 Ab	< 0.2	< 0.2 U
CK	18	< 200 U/L
ESR	**93**	< 30 mm/h
CRP	**4.3**	< 0.9 mg/dL
Infectious workup	Result	Normal
Anti-HCV IgG Ab	Negative	Negative
HCV RNA PCR	Negative	Negative
HBV surface Ag	Negative	Negative
HIV Ab screen	Negative	Negative
ASO Ab screen	26	< 300 U/mL
Hematologic workup	Result	Normal
Free κ light chains	**5.8**	0.3 – 2 mg/dL
Free λ light chains	**1.7**	0.6 – 2.6 mg/dL
Free κ/λ light chain ratio	**3.4**	0.3 – 1.6
Serum protein immunofixation electrophoresis	**2 g/dL IgG-**κ	Negative
Urine protein immunofixation electrophoresis	**IgG-**κ	Negative
Cryoglobulins	< 1 (24 h), 1 (72 h)	0 – 1%

Anti-CCP Ab = anti-cyclic citrullinated peptide antibody; anti-LA Ab = anti-La Ab; anti-RO Ab = anti-ro Ab; anti-RF antibody = anti-rheumatoid factor; C3 = complement component 3; C4 = complement component 4; CK = creatine kinase; ESR = erythrocyte sedimentation rate; CRP = C-reactive protein; HCV IgG = hepatis C virus immunoglobulin G; RNA PCR = ribonucleic acid polymerase chain reaction; HBV surface Ag = hepatitis B virus surface antigen; HIV = human immunodeficiency virus; ASO = antistreptolysin O.

**Table 2.** A brief review of renal pathologies associated with paraproteinemias.

Pathology dependent on high Ig-burden. High likelihood of symptomatic myeloma.	
Cast nephropathy “myeloma kidney”	Most common AKI in MM and a MM defining event. A high burden of filtered LCs form tubular casts/crystals obstructing the distal nephron. Hypercalcemia is also common. An indication for urgent chemotherapy while plasmapheresis is controversial.
Waldenstrom’s macroglobulinemia	Rare. Monoclonal IgM form glomerular intracapillary thrombi as part of hyperviscosity syndrome.
Pathology dependent on the structural pathogenicity of Ig. Low likelihood of symptomatic myeloma with generally lower tumor burden. Can be seen in myeloma or MGUS/MGRS.	
Monoclonal immunoglobulin deposition disease (MIDD)	Presents with proteinuria, CKD, ± nephrotic syndrome. Filtered Ig (light and/or heavy chains) deposit in GBM and TBM causing thickening. Vasculature may be involved. Nodular mesangial sclerosis seen in 2/3 and associated with nephrotic range proteinuria. LCDD is mostly κ. Only 20% have symptomatic myeloma at diagnosis.
Amyloid	Presents with proteinuria, CKD, ± nephrotic syndrome. AL more common than AH or AHL. 75% λ in AL. β-pleated sheets of Ig deposit in glomeruli, GBM, tubules, and vasculature. Fibrils are organized, non-branching, 7 – 14 nm, with + Congo-red stain. TBM thickness usually normal. Less than 10% symptomatic myeloma at diagnosis but extra-renal involvement frequent (e.g., cardiac, hepatic, and peripheral neuropathy). Patients may be hypotensive with altered renal autoregulation.
Glomerulonephritis	Rare. Presents with hematuria ± nephritic or nephrotic syndrome. Diagnosis based on pathology with multiple possibilities including immunotactoid GN, type 1 cryoglobulinemic GN, proliferative GN with monoclonal Ig deposits, C3 GN.
Fanconi’s syndrome	Monoclonal Ig inclusions in the proximal tubule, with or without crystals. May be isolated or present as part of other pathologies such as amyloid or MIDD.
Renal disease unrelated to immunoglobulin	
Decreased renal perfusion	Hypercalcemia, hypovolemia, sepsis.
Medications	NSAIDs and pamidronate (FSGS, ATN).
TLS	Rare
Lymphoma or plasma cell infiltration	Rare

Ig = immunoglobulin; AKI = acute kidney injury; MM = multiple myeloma; LCs = light chains; IgM = immunoglobulin M; MGUS/MGRS = monoclonal gammopathy of unknown significance, monoclonal gammopathy of renal significance; CKD = chronic kidney disease; GBM = glomerular basement membrane; TBM = tubular basement membrane; LCDD = light chain deposition disease; AL = light chain amyloidosis; AH = heavy chain amyloidosis; AHL = heavy and light chain amyloidosis; nm = nanometers; GN = glomerulonephritis; C3 = complement component 3; NSAIDs = non-steroidal anti-inflammatory drugs; FSGS = focal-segmental glomerulosclerosis; ATN = acute tubular necrosis; TLS = tumor lysis syndrome.
